# Screening for mismatch repair deficiency in colorectal cancer: data from three academic medical centers

**DOI:** 10.1002/cam4.1025

**Published:** 2017-05-03

**Authors:** Grainne M. O'Kane, Éanna Ryan, Terri P. McVeigh, Ben Creavin, John MP. Hyland, Diarmuid P. O'Donoghue, Denise Keegan, Robert Geraghty, Delia Flannery, Carmel Nolan, Emily Donovan, Brian J. Mehigan, Paul McCormick, Cian Muldoon, Michael Farrell, Conor Shields, Niall Mulligan, Michael John Kennedy, Andrew J. Green, Desmond C. Winter, Padraic MacMathuna, Kieran Sheahan, David J. Gallagher

**Affiliations:** ^1^ St. James's HospitalDublin 8Ireland; ^2^ Centre for Colorectal DiseaseSt. Vincent's University HospitalDublin 4Ireland; ^3^ Department of Clinical GeneticsOur Lady's Children's HospitalDublin 12Ireland; ^4^ Mater Misericordiae University HospitalDublin 7Ireland

**Keywords:** Colorectal cancer, mismatch repair deficiency, reflex immunohistochemistry, screening

## Abstract

Reflex immunohistochemistry (rIHC) for mismatch repair (MMR) protein expression can be used as a screening tool to detect Lynch Syndrome (LS). Increasingly the mismatch repair‐deficient (dMMR) phenotype has therapeutic implications. We investigated the pattern and consequence of testing for dMMR in three Irish Cancer Centres (CCs). CRC databases were analyzed from January 2005–December 2013. CC1 performs IHC upon physician request, CC2 implemented rIHC in November 2008, and CC3 has been performing rIHC since 2004. The number of eligible patients referred to clinical genetic services (CGS), and the number of LS patients per center was determined. 3906 patients were included over a 9‐year period. dMMR CRCs were found in 32/153 (21%) of patients at CC1 and 55/536 (10%) at CC2, accounting for 3% and 5% of the CRC population, respectively. At CC3, 182/1737 patients (10%) had dMMR CRCs (*P* < 0.001). Additional testing for the *BRAF* V600E mutation, was performed in 49 patients at CC3 prior to CGS referral, of which 29 were positive and considered sporadic CRC. Referrals to CGS were made in 66%, 33%, and 30% of eligible patients at CC1, CC2, and CC3, respectively. LS accounted for CRC in eight patients (0.8%) at CC1, eight patients (0.7%) at CC2, and 20 patients (1.2%) at CC3. Cascade testing of patients with dMMR CRC was not completed in 56%. Universal screening increases the detection of dMMR tumors and LS kindreds. Successful implementation of this approach requires adequate resources for appropriate downstream management of these patients.

## Introduction

Lynch Syndrome (LS) is a common cancer predisposition syndrome caused by germ‐line mutations in genes involved in the mismatch repair (MMR) pathway (*MLH1*,* MSH2*,* MSH6*,* PMS2,* and *EPCAM*). A defective MMR system results in the hypermutable phenotype of microsatellite instability (MSI) and drives carcinogenesis 1. Colorectal cancer (CRC) is the most prevalent LS associated malignancy accounting for the highest mortality rates in this population 2. Approximately 2–4% of all CRCs can be attributed to LS 3, 4, and in patients <35 years this increases to 12% 5. Although the true incidence remains unknown, LS likely represents one of the most common Mendelian conditions 6. Surveillance colonoscopy 2, 7, prophylactic gynecologic surgery 8, and total colectomy at CRC diagnosis 9, 10, 11, 12 have the potential to reduce mortality and morbidity in this population. Medical prevention with aspirin has been shown to reduce the incidence of CRC in LS carriers 13, and may be offered to patients as a chemopreventative agent 14.

Screening all incident CRC diagnoses (universal screening) for microsatellite instability (MSI) via PCR or reflex IHC (rIHC) for dMMR is increasingly undertaken at many academic institutions to determine adjuvant treatment 15, and to predict response to chemotherapeutic agents^16^, ^17^, ^18^ and immune‐checkpoint inhibitors 19 20.

The ability to identify LS and the potential therapeutic implications support the rationale for universal screening. Universal testing has not been adopted worldwide. Appropriate resourcing is required to support screening programs, and the requirement for patient consent is debated 21, 22.

Historically, screening had been driven by the use of clinical guidelines based on age and family history, such as the Amsterdam 23, 24 and Bethesda criteria 25, 26. However, it has been shown that up to 50% of mutation carriers do not fulfill the Amsterdam criteria and 40–45% of families fulfilling these criteria do not have MSI on tumor testing 27, 28. The Bethesda guidelines incorporate tumor histopathologic features and while more sensitive, can still miss 12–28% of LS cases 4, 29, 30, 31, 32. The Evaluation of Genomic Applications in Practice and Prevention group (EGAPP) 33 were the first group to recommend universal screening. These were followed by recommendations of a selective screening strategy using 70 years as an age cutoff (The Jerusalem criteria) 34. This method was estimated to have a sensitivity of only 85% 4. The US Multi‐Society Taskforce on Colorectal Cancer (MSTC) and the National Comprehensive Cancer Network (NCCN) have recommended either universal screening or selective screening in patients ≤70 years and >70 years with a family history concerning for LS 35, 36. The American Society for Clinical Pathology, the College of American Pathologists (CAP), the Association for Molecular Pathology, and the American Society of Clinical Oncology (ASCO) have recently drafted joint guidelines on CRC molecular testing suggesting universal testing in all CRCs for prognostic stratification and identification of Lynch syndrome patients 37. Both IHC and/or MSI testing are endorsed screening tools. Algorithms have suggested the incorporation of B‐RAF proto‐oncogene serine/threonine kinase (*BRAF)* mutation and/or MLH1 hypermethylation testing to exclude sporadic disease prior to referral to clinical genetic services (CGS)^33^, ^35^.

We investigated dMMR detection rates by IHC and subsequent downstream testing at three Irish Cancer Centres (CCs).

## Methods

### Study population

Cancer care within Ireland is centralized at eight CCs. Each CC has an individual pathology department that can perform IHC for MMR proteins. The availability of resources to complete downstream LS testing is variable at each CC. Generally, an external referral to CGS is required for genetic testing. There are currently no national guidelines on universal screening for LS.

New CRCs at three academic institutions diagnosed between January 2005 and December 2013 were reviewed. During the studied period approaches to screening using IHC differed at each CC. CC1 performed IHC at the request of the physician. CC2 began universal screening using rIHC in November 2008. Neither of these sites performed *BRAF* testing prior to CGS referral. CC3 has been using rIHC as a screening tool since 2004 and has maintained a prospective database of clinicopathologic features. Since 2012, this center has added *BRAF* mutation testing to their screening protocol in MLH1*‐*deficient tumors. Incident CRCs at each CC are discussed at weekly institutional multidisciplinary team (MDT) meetings.

### dMMR detection rates and CGS referral

Patients were identified from CRC databases at each center and pathology reports reviewed. Only patients treated at the institution with available clinical information were included. Histologies other than adenocarcinoma, low‐grade appendiceal adenocarcinomas, and small bowel adenocarcinomas were excluded from the analysis. Synchronous or metachronous CRCs were documented as separate specimens but as a single patient. The number of specimens with available IHC and the percentage of tumors exhibiting MMR protein loss were recorded. In cases where IHC identified MLH1 protein loss and additional *BRAF* testing was positive for a V600E mutation, the CRC was considered sporadic, and such patients were presumed ineligible for referral to CGS unless otherwise indicated. At CC3, prior to the introduction of *BRAF* testing in MLH1‐deficient cancers, the absence of a family history of CRC in patients older than 70 years was used to separate sporadic and potential LS cases and whether downstream testing was performed.

Electronic patient records, patient letters, and institutional familial cancer databases were evaluated for referrals to CGS. All dMMR patients were cross‐referenced for attendance at the CGS in CC1 and CC2, or the Department of Clinical Genetics, Our Lady's Children's Hospital, where national genetic testing is processed. The number of patients who were referred for a genetic medicine consultation, and those who subsequently consented to germ‐line testing were recorded. Individual mutations were documented where possible.

### Statistical analysis

Information related to patient numbers and demographics are presented using descriptive statistics. Data for continuous variables are reported as median and range. Data for qualitative variables are reported as percentages. Differences in the numbers of patients with available IHC and numbers of dMMR patients detected were analyzed using the chi‐squared test. Statistical analysis was performed using GraphPad Prism 7 (GraphPad software, California La Jolla, USA).

## Results

### dMMR detection using IHC

A total of 3906 patients across the three centers over a 9‐year period were reviewed. The median age at CRC diagnosis was 70 years at CC1 and 2, and 69 years at CC3. At CC1 (IHC performed at physician request) 153/949 (16%) patients had tumor MMR testing of which 32 (21%) were dMMR accounting for 3% of the CRC population included. CC2 (implemented rIHC in November 2008) performed testing on 536/1220 (44%) patients of which 55(10%) had dMMR tumors resulting in a detection rate of 5% (*P* = 0.22). Post rIHC implementation, the number of samples with available results at CC2 was 67%. The primary reason for absent reporting was “insufficient tumor for analysis.” Diagnostic biopsies in patients with stage IV disease, T1 lesions or complete responses to neoadjuvant therapy represented 88% of these. CC3 completed rIHC on 100% of specimens and determined that 10% (182/1737) of patients had dMMR CRCs (*P* < 0.001). The most common pattern of MMR protein loss at all sites was MLH1+/‐ PMS2 (Table 1.).

**Table 1:** IHC availability and dMMR detection rates per CC

	Centre 1 (IHC when requested)	Centre 2 (rIHC since Nov 2008)	Centre 3 (rIHC since 2004)
Total new CRC with available histology N=3963	964	1246	1753
Total patient number N=3906	949	1220	1737
Median age yrs. (range)	70 (17–97)	70 (16–97)	69 (26–96)
No. (%) patients with IHC	153 (16%)	536(44%)	1737 (100%)
No. (%) patients with MMR‐d	32 (3%)	55 (5%)	182 (10%)
Median age years. (range)	62 (24–85)	75 (35–94)	75 (28–93)
No. (%) BRAF testing pre GS	0	0	49 (27%) 29 +ve 20 WT
MLH1/PMS2	14 (44%)	34 (62%)	158 (87%)
MSH2/MSH6	4 (12%)	5 (9%)	18 (10%)
MLH1	9 (28%)	13 (24%)	0
MSH2	4 (12%)	0	0
MSH6	1 (4%)	1 (2%)	4 (2%)
PMS2	0	2 (4%)	2 (1%)

### Downstream management of abnormal dMMR results

**Figure 1:**
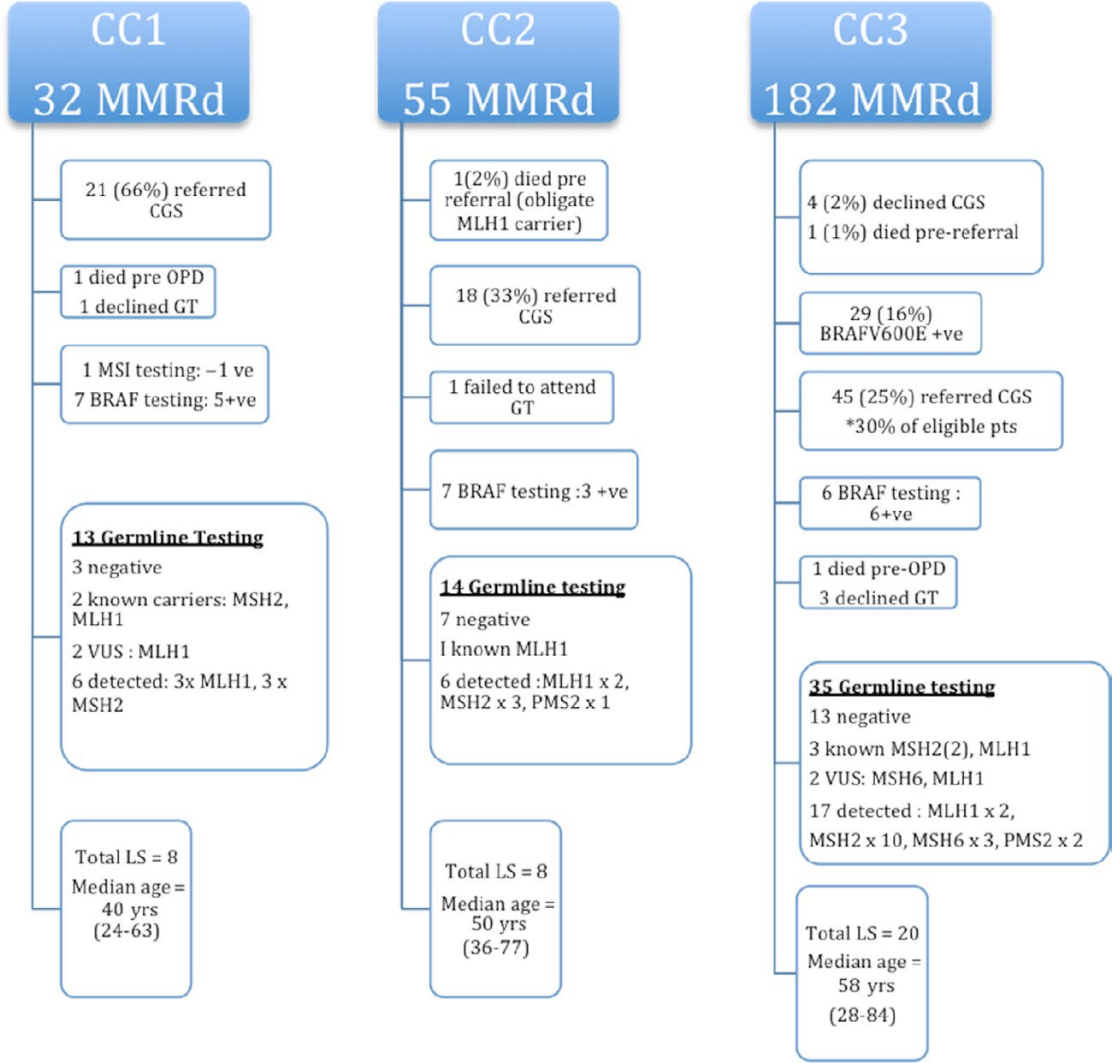
Flow diagram showing downstream work‐up of patients with dMMR CRC.

Figure 1 depicts the workflow of patients from abnormal dMMR result to LS detection.

Testing for the *BRAF*V600E mutation was not performed at CC1 or CC2 prior to CGS referral.

The median age of the dMMR cohort (*N* = 32) at CC1 was 61.5 years. (24–85 years.) Twenty‐one (66%) patients were referred to CGS. One patient died pre appointment and one declined germ‐line testing. Another patient had normal tumor MSI testing and did not undergo further work‐up. Five of seven patients tested had a positive *BRAF*V600E mutation in tumor (median age 82 years., range 58–85 years), and these patients did not undergo any further investigations. The remaining 13 patients had germ‐line testing. This includes two known carriers who had predictive testing prior to CRC diagnosis. There were no MMR gene mutations found in three patients and two variants of unknown significance (VUS) were identified. Six LS patients were newly diagnosed. Overall, eight LS patients (median age 40 years, range 24–63 years) were present in the CRC population (0.8%).

The median age of the dMMR (*N* = 55) cohort at CC2 was 75 years (35–94 years.), One patient died pre referral but was an obligate *MLH1* carrier. Eighteen patients (33%), seven pre rIHC and 11 post rIHC, were referred to CGS. One patient failed to attend for germ‐line testing. Seven patients had tumors tested for the *BRAF*V600E mutation of which three were positive (ages 61, 71, and 80 years.). Fourteen (14/18) patients underwent germ‐line testing, including one known *MLH1* carrier. Results were negative in seven patients and LS was diagnosed in six. Overall LS accounted for CRC in eight patients (median age 50 years, range 36–77 years) at CC2 (0.7%).

At CC3 the median age of the 182 dMMR patients was 75 years. (28–93 years.). Four patients (2%) declined CGS referral. One patient (<1%) died pre CGS referral. Additional *BRAF* mutation analysis was performed in 55 of the overall cohort of 158 patients with tumors exhibiting MLH1 loss. Sixty‐four percent (35/55) were positive for the *BRAF*V600E mutation. The median age of patients in this group was 78 years. Of these 35, 29 had testing pre CGS and presumed to have sporadic disease. In total 45/182 (25%) patients or 45/148 eligible patients (30%) were referred to CGS. A further six patients had tumors with the *BRAF*V600E mutation detected post CGS referral. Three patients declined germ‐line analysis and one died pre‐appointment. Thirty‐five patients had germ‐line testing, including three known carriers. Wild‐type results returned 13 patients and a VUS was identified in two patients. Mutations in MMR genes were identified in 17 patients (median age 58 years, range 28–84 years). Overall LS accounted for CRC in 20 patients (1.2%).

### Characteristics of patients not referred to CGS

In total, 150/268 (56%) patients with dMMR CRC were not referred to CGS or declined further testing. The majority (92%; *n* = 139) of these were individuals who had tumors exhibiting MLH1 +/‐ PMS2. Of these, 71% (*n* = 107) were greater than 70 years old. Two patients had isolated tumor PMS2 loss (ages 68 and 86 years). Nine patients had either MSH2 + MSH6 loss or MSH6 loss only (median 67.5 years. Range 55–88 years). Where possible, these patients have now been contacted and invited to attend a clinical genetics appointment.

## Discussion

In this retrospective multicenter study, universal screening for dMMR using IHC was feasible and resulted in an increased number of dMMR CRCs. However, 56% of all dMMR patients tested at CC1‐CC3 either were not referred to CGS, declined further testing or did not have additional downstream *BRAF* or MLH1 hypermethylation testing performed. The feasibility and effectiveness of universal screening has previously been reported 29, 38. Practical challenges in implementation and concerns regarding real world cost‐effectiveness have been highlighted 21, 22. Although pathologists are considered the “driving force” of universal screening 39 uncertainty remains as to who is responsible for management of results 40. Clinician awareness and timely access to services are reported referral barriers 41. When a surgeon received dMMR results 55% of patients were subsequently referred to CGS. When both a genetic counselor and surgeon received results, and the genetic counselor was able to facilitate the referral, this increased to 100% 38. Referrer education, timely access to expert opinion and an appropriately resourced infrastructure are required for successful incorporation of clinically relevant molecular testing cascades into everyday practice.

The majority of patients with incomplete downstream investigations were >70 years with tumor loss of MLH1 expression 42. *BRAF* mutation testing in this phenotype can often identify patients with sporadic tumors owing to *MLH1* promoter methylation 43. These patients do not usually require further additional testing. In this study 69 patients had *BRAF* testing performed of which 43 (62%) harbored mutation; 77% of these patients were >70 years. CGS ordered *BRAF* testing in 11/43 (26%) patients.

By classifying tumors as sporadic in this way, first degree relatives of patients with unstable tumors do not require routine screening, efficiency is improved and costs reduced 44 45, 46, 47. *MLH1* promoter hypermethylation testing may be incorporated sequentially into this approach or may be superior to *BRAF* as an initial test in determining the origin of MLH1 hypermethylation in MLH1‐deficient tumors 48. Additional data are needed on whether both *BRAF* and *MLH1* hypermethylation testing are required. Clinical suspicion and resource availability contribute to extent of testing in clinical practice. Institutions implementing reflex screening using IHC, should incorporate additional testing to exclude sporadic tumors.

Most new LS cases identified in this study were ≤70 years at CRC diagnosis, median 49 years (range 24–84 years). Four of 36 (11%) LS cases were >70 years and only one of these patients did not have a documented family history of malignancy of CRC. LS patients over 70 years presenting with CRC in the absence of a positive family history appear uncommon 3. The median age of dMMR patients at CC2 and CC3 was 75 years. The median age at CC1 was 62 years reflecting the use of clinical criteria to select a younger dMMR population. At this center 21% of tumors tested were dMMR, which corresponds with the sensitivity of the Amsterdam Criteria 3. The specificity of IHC in detecting LS patients decreases with age 42. Moreira et al. showed that universal screening of all CRCs was the most sensitive method in detecting LS. An alternative approach of screening patients ≤70 years or >70 years meeting Bethesda guidelines was the next most sensitive method. This missed only 5% of cases but resulted in 35% less tumor testing 4. While universal testing may be the optimal approach, population screening with an age cut‐off would reduce downstream investigations, and limit the associated societal costs of testing 45.

A number (11%) of patients declined or did not attend for counseling and/or germ‐line testing after referral to CGS. This represents a higher uptake than previously reported, where up to 50% declined germ‐line testing 31, 49. A small number of patients died before referral or attendance at CGS. This represents a challenge 50, 51, 52, 53 and may require discussion of LS work‐up with next of kin 54. Thirty‐eight percent of patients received wild‐type genetic test results. Screening for relatives of these individuals is dictated by family history, and CRC screening recommendations are generally often similar to those with confirmed LS 35. Patients with wild‐type test results, referred to as “Lynch‐Like” Syndrome (LLS), have an uncertain phenotype. Bi‐allelic somatic inactivation of MMR genes accounts for over 50% of cases 55. Recently somatic mutations in *POLE* have proven causative of dMMR in these patients 56. The complexity of downstream testing may support upfront germ‐line testing.

In a small country with a national healthcare system testing strategies vary between pathology departments. Universal IHC testing of all samples was not completed in CC2, due largely to the size of available samples. Clinical criteria such as age and family history can be used to direct genetic testing where tissue is unavailable 36, 57. However, the use of clinical criteria alone seems to result in too small a referral number as evidenced in CC1. The feasibility and uptake of universal screening within countries has been reported previously 58, 59, 60. IHC is subject to operator skill and studies have shown inter‐observer variability. Testing should therefore be performed only in expert settings, where high quality control measures are in place 35, 61, 62. Questions remain regarding the education of key stakeholders, consent, the impact on already overstretched practitioners and/or institutions, and the lack of access to CGS 63. Clear and concise guidelines must be continually developed, even within health systems or institutions that have already established screening programs, in accordance with international best practice and the rapid pace of change in this emerging area 21. Investment in infrastructure and testing costs would facilitate uniformity in practice, resulting in a safer, higher quality and ultimately cost‐saving approach, that would be applicable not just to LS but to the incorporation of genomic medicine into routine practice 63.

There are a number of limitations to this study. This is a retrospective analysis, which includes a time period when universal IHC was not part of routine guidelines, and BRAF testing/hypermethylation testing was not readily available. We were unable to determine the impact of CGS access or availability, but the limited resources available in Ireland may provide a barrier to accessing services 64. The number of LS patients at CC3 accounted for 1.2% of all CRCs and is likely an underestimate of the true Lynch syndrome rate. This may be due to the unavailability of *BRAF*/MLH1 hypermethylation testing in the early part of this study, or due to the inability of IHC to detect missense mutations 65. The lack of uniform practice in this study prevented identification of a true national rate of LS diagnosis.

In conclusion, interest in determining the MMR status of CRC has grown due to its increased clinical utility. Universal screening using rIHC is feasible, however successful implementation requires adequate funding, including protocols to manage all downstream dMMR results. Alternative strategies such as upfront germ‐line testing and the use of next generation sequencing determine the mutational load of a tumor may obviate the need for MMR/MSI testing.

We have demonstrated the complexity of incorporating a simple and robust testing pathway into clinical practice. Successful universal adoption of this paradigm would provide a model for the interpretation and utilization of complex genomic test results.

## Conflict of Interest

The authors have no disclaimers.
